# Beneficial Effects of Calcitriol on Hypertension, Glucose Intolerance, Impairment of Endothelium-Dependent Vascular Relaxation, and Visceral Adiposity in Fructose-Fed Hypertensive Rats

**DOI:** 10.1371/journal.pone.0119843

**Published:** 2015-03-16

**Authors:** Chu-Lin Chou, Cheng-Yoong Pang, Tony J. F. Lee, Te-Chao Fang

**Affiliations:** 1 Institute of Medical Sciences, Tzu Chi University, Hualien, Taiwan; 2 Institutes of Life Sciences, Pharmacology & Toxicology, and Medical Sciences, Tzu Chi University, Hualien, Taiwan; 3 Department of Pharmacology, Southern Illinois University School of Medicine, Springfield, Illinois, United States of America; 4 Division of Nephrology, Department of Internal Medicine, Wan Fang Hospital, Taipei Medical University, Taipei, Taiwan; 5 Department of Internal Medicine, School of Medicine, College of Medicine, Taipei Medical University, Taipei, Taiwan; Institut National de la Santé et de la Recherche Médicale, FRANCE

## Abstract

Besides regulating calcium homeostasis, the effects of vitamin D on vascular tone and metabolic disturbances remain scarce in the literature despite an increase intake with high-fructose corn syrup worldwide. We investigated the effects of calcitriol, an active form of vitamin D, on vascular relaxation, glucose tolerance, and visceral fat pads in fructose-fed rats. Male Wistar-Kyoto rats were divided into 4 groups (n = 6 per group). Group Con: standard chow diet for 8 weeks; Group Fru: high-fructose diet (60% fructose) for 8 weeks; Group Fru-HVD: high-fructose diet as Group Fru, high-dose calcitriol treatment (20 ng / 100 g body weight per day) 4 weeks after the beginning of fructose feeding; and Group Fru-LVD: high-fructose diet as Group Fru, low-dose calcitriol treatment (10 ng / 100 g body weight per day) 4 weeks after the beginning of fructose feeding. Systolic blood pressure was measured twice a week by the tail-cuff method. Blood was examined for serum ionized calcium, phosphate, creatinine, glucose, triglycerides, and total cholesterol. Intra-peritoneal glucose intolerance test, aortic vascular reactivity, the weight of visceral fat pads, adipose size, and adipose angiotensin II levels were analyzed at the end of the study. The results showed that the fructose-fed rats significantly developed hypertension, impaired glucose tolerance, heavier weight and larger adipose size of visceral fat pads, and raised adipose angiotensin II expressions compared with the control rats. High- and low-dose calcitriol reduced modestly systolic blood pressure, increased endothelium-dependent aortic relaxation, ameliorated glucose intolerance, reduced the weight and adipose size of visceral fat pads, and lowered adipose angiotensin II expressions in the fructose-fed rats. However, high-dose calcitriol treatment mildly increased serum ionized calcium levels (1.44 ± 0.05 mmol/L). These results suggest a protective role of calcitriol treatment on endothelial function, glucose tolerance, and visceral adiposity in fructose-fed rats.

## Introduction

Vitamin D has been used as a therapy for rickets since 1919 [1]. Besides classically regulating calcium and bone health, vitamin D has also been reported to play a role in cardiovascular and metabolic diseases [2, 3]. For example, observational studies have linked low 25-hydroxyvitamin D levels to higher blood pressure [4, 5] and cardiovascular disease [3, 6–8]. In addition, increased risks of diabetes and obesity have been reported among persons with low 25-hydroxyvitamin D levels [3, 9, 10]. However, data on whether vitamin D supplementation can lessen the progression of cardiovascular and metabolic diseases are lacking.

Glucose intolerance has increased worldwide, and this has been linked to the increased intake of high-fructose corn syrup [11]. A high-fructose diet (60% fructose) in rodents has been reported to result in raised blood pressure, glucose intolerance, and hyperlipidemia as well as exacerbating the activity of the renin-angiotensin system (RAS) [12–15]. Thus, animal models of fructose-fed rats have commonly been used in the investigation of hypertension and metabolic disturbances.

Several studies have suggested that vitamin D plays a regulatory role in the cardiovascular system through down-regulation of RAS by directly inhibiting renin transcription [16, 17]. Recently, calcitriol (1, 25-dihydroxyvitamin D_3_, a major active form of vitamin D) supplementation has been reported to improve aortic vascular tone and cardiac hypertrophy in spontaneously hypertensive rats (SHR) [18–20]. However, no experimental studies have yet examined the effect of calcitriol on vascular dysfunction and metabolic disturbances in fructose-fed rats. Therefore, the aim of this study was to evaluate the efficacy of calcitriol on reducing systolic blood pressure, improving glucose intolerance, restoring the ability of endothelium-dependent vascular relaxation, and shrinking enlarged visceral fat pads weights and adipose sizes in fructose-fed rats.

## Methods

### Animals

All experimental procedures were carried out with the prior approval of the Institutional Animal Care and Use Committee of Tzu Chi University (Permit number: 101-28) and in strict accordance with the recommendations in the Guide for the Care and Use of Laboratory Animals of the National Institutes of Health. All surgeries were performed under sodium pentobarbital anesthesia, and all efforts were made to minimize suffering. Male Wistar-Kyoto rats, initially weighing 200 to 230 g, were used for the experiments. Rats were placed in individual cages and kept in a room with air maintained at a temperature of 24–27°C, a humidity of 50–80%, and a 12 h light/dark cycle, and had access to tap water ad libitum throughout the experiments.

The high-fructose diet (Harlan Teklad, Madison, WI) was composed of 60% fructose, 21% protein, 5% fat, 8% cellulose, and standard vitamins and mineral mix. The standard chow diet was composed of 50% starch, 21% protein, 4% fat, 4.5% cellulose, and standard vitamins and mineral mix.

### Experimental Protocols

After an adaptation period of 1 week, the male Wistar-Kyoto rats were divided into 4 groups (n = 6 for each group) and the study was conducted for 8 weeks. The rats were fed as follows: Group Con: standard chow diet for 8 weeks, served as the control group; Group Fru: high-fructose diet (60% fructose) for 8 weeks; Group Fru-HVD: the same high-fructose diet as Group Fru, and high-dose calcitriol (20 ng / 100 g body weight per day) via a subcutaneous osmotic minipump was administered 4 weeks after the beginning of fructose feeding; and Group Fru-LVD: the same high-fructose diet as Group Fru, and low-dose calcitriol (10 ng / 100 g body weight per day) via a subcutaneous osmotic minipump was administered 4 weeks after the beginning of fructose feeding. The dosage of calcitriol was based on that used in previous studies [18, 21] which showed that calcitriol treatment with less than 25 ng / 100 g per day did not cause serum calcium increases and urine calcium excretions [22], and vascular calcifications in rats with intact renal function [23]. The first day of fructose feeding was defined as Day 1. Body weight was measured twice a week, and systolic blood pressure (SBP) was also measured twice a week by the tail-cuff method. Blood samples (1 mL) for levels of serum triglycerides, total cholesterol, glucose, ionized calcium, phosphate, and creatinine were taken after 12 h of fasting at Day 0, Day 28 and Day 56 from the femoral vein using a indwelling catheter once at that time under sodium pentobarbital anesthesia (40 mg/kg, intraperitoneal injection). Serum was separated and divided into aliquots and frozen until analysis.

### SBP Measurements

The rats were removed from the animal room and taken to the laboratory at 8 AM. Rats were allowed free access to water and were kept in a quiet area before SBP was measured at 9 AM. The tail-cuff method without preheating was used to reliably measure SBP with a programmed electro-sphygmomanometer (MK-2000ST, Muromachi, Tokyo, Japan), as our previous studies [14, 24–26] and other studies [27–30]. The mean of 6 consecutive readings was used as the value of SBP for each rat for that day, and SBP was determined twice a week during the adaptation (1 week) and experimental (8 weeks) periods.

### Osmotic Minipump Installation

The osmotic minipump installation was performed as described in our previous studies [14, 24–26]. In brief, an osmotic minipump (No. 2002, 14 days of active life, Alza Corp) was filled with calcitriol (Abbott Laboratories, Green Oaks, IL, USA), which was dissolved in propylene glycol (No.9402, J.T. Baker, Philipsburg, NJ, USA), and implanted subcutaneously in the rats under brief anesthesia with sodium pentobarbital (40 mg/kg, IP). Aqueous penicillin (5000 U/kg, subcutaneous injection) was administered immediately after minipump implantation. At the end of the life of the minipump, a new one was implanted and the used one removed. The residual volume in each minipump removed from the rats was carefully examined to ensure that the minipump release function was normal.

### Intra-peritoneal Glucose Tolerance Test (IGTT)

An IGTT was performed on Day 55. After 12 h of fasting, 0.1 mL of blood was taken from the tail nick that rat tail was only cut once, such as other studies [31–33], and serum glucose was measured using an Accu-Check Advantage Blood Glucose Monitor (Roche Diagnostic Corporation, Indianapolis, IN, USA). Immediately after this baseline measurement, a dose of 50% glucose solution (1 g/kg body weight) was injected intraperitoneally as described previously [34]. The serum glucose level was measured at 0, 30, 60, 90, and 120 minutes after the glucose load.

### Laboratory Measurements

The blood samples were immediately centrifuged at 4000 × *g* at 4°C for 10 minutes. The serum samples were separated and used immediately for assays of glucose, triglycerides, and total cholesterol. Serum triglycerides, total cholesterol, glucose, ionized calcium, phosphate, and creatinine were determined by standard methods using a COBAS Integra 800 analyzer (Roche Diagnostics, Indianapolis, IN, USA). Serum insulin was measured using a commercial rat enzyme immunoassay kit (EZRMI-13K, Millipore, St Charles, MO, USA). Serum 1,25-(OH)_2_D_3_ was determined by a commercial enzyme immunoassay (AC-62F1, ImmunoDiagnosticSystems, UK) [35].

### *In Vitro* Vascular Reactivity

*In vitro* vascular reactivity was assessed as described in our previous studies [14, 36]. The isolated rings of the thoracic aortas were obtained from the four groups of rats for analysis of endothelium-dependent vascular contraction at Day 56. The thoracic aortas were dissected and aortic ring segments (3 mm in length) were suspended in individual organ chambers filled with oxygenated (95% O2 and 5% CO2) Krebs’ buffer solution maintained at 37°C. Krebs solution consisted of 117 mmol/L NaCl, 25 mmol/L NaHCO3, 4.7 mmol/L KCl, 2.5 mmol/L CaCl2, 1.2 mmol/L MgSO4, 1.2 mmol/L KH2SO4, 11.1 mmol/L glucose, and 0.28 mmol/L ascorbic acid at pH 7.4. Tension changes in the aortic rings were recorded using an isometric transducer (FT03C; Grass, West Warwick, RI, USA) connected to a Grass polygraph. A resting tension of 2 g was applied to the aortic ring, and the active muscle tone of ring segments was then appropriately contracted by phenylephrine. After a stable contraction plateau was reached, relaxation of the aortic rings was measured in response to cumulative additions of acetylcholine (ACh, Sigma; 10^−9^ to 10^−4^ mol/L). After the last vasodilator response to acetylcholine, a single dose of NG-nitro-L-arginine methyl ester (L-NAME; 100 nmol), a nitric oxide (NO) synthesis inhibitor, was applied. When a steady contraction in response to phenylephrine and L-NAME was reached, the vasodilator response to sodium nitroprusside (SNP, Riedel-deHaen, Seelze, Germany; 10^−9^ to 10^−4^ mol/L) was evaluated.

### Measurement of the Weight of Visceral Fat Pads and Adipocyte Sizes

Visceral fat pads were obtained at Day 56. Retroperitoneal, mesenteric, and epididymal fat pads were rapidly dissected, weighed and stored at −80°C. The weights of the fat pads were measured and normalized to body weight (gram/gram, %), and 3-mm thick paraffin-embedded specimens of the fat pads were stained with hematoxylin–eosin. Adipocyte sizes were photographically measured and counted under a microscope. An average of 50 measurements of adipocyte size (diameter, μm) was taken as each individual value according to the method of Furuhashi *et al*. [37]

### Analysis of Adipose Angiotensin II (Ang II) Levels

To determine the levels of Ang II derived from the retroperitoneal, mesenteric, and epididymal fat pads, adipose samples (0.3 mg) were homogenized at 4°C in tissue extraction buffer (0.6 mL) for 15 minutes. The tissue extraction buffer was a commercially available buffer consisting of 29.4 mL T-PER tissue protein extraction reagent (Thermo Scientific, USA), 300 μL Halt protease and phosphatase inhibitor cocktail (Thermo Scientific, USA) and 300 μL EDTA. After freezing with liquid nitrogen for five seconds, adipose samples were centrifuged at 10,000 × g for 20 minutes. After centrifugation, 0.5 mL of the supernatant (below the fat layer) was assayed. For adipose Ang II levels, 0.5 mL of the supernatant was measured by a quantitative sandwich enzyme immunoassay technique using a commercially available Ang II kit (SPI Bio, Montigny le Bretonneux, France) following the manufacturer’s instructions. Tissue protein quantitation was measured using a Pierce BCA Protein Assay Kit (Thermo Scientific, USA). Adipose Ang II values were expressed as picogram.mg^−1^ of protein.

### Statistical Analysis

All results were expressed as means ± SD. Experimental data over time were compared between groups by two-way analysis of variance (ANOVA), the first factor being treatment group, and the second the time period. When a significant effect was detected by ANOVA, the Newman-Keuls test was used to establish statistically significant differences between means. The Student's t-test for unpaired data was also performed when appropriate. A *P* value less than 0.05 was considered to be statistically significant.

## Results

### Calcitriol Ameliorated Systolic Hypertension in the Fructose-fed Rats

**Fig 1:**
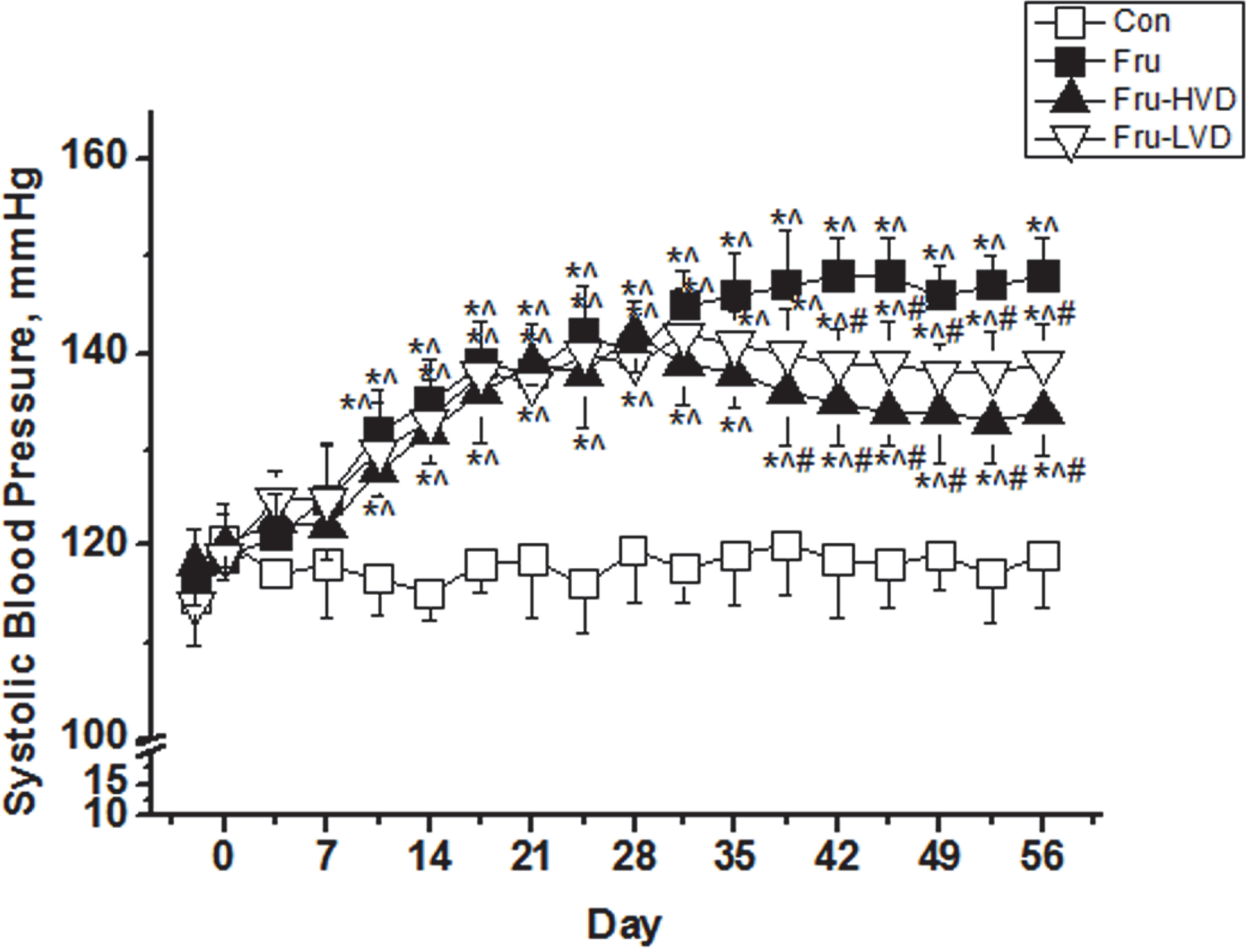
Changes in systolic blood pressure (SBP) in the control and fructose-fed rats with and without calcitriol treatment. Con: control rats with normal chow diet; Fru: rats were fed a high-fructose diet for 8 weeks; Fru-HVD: rats were treated as Group Fru, and high-dose calcitriol (20 ng / 100 g body weight per day) was administered 4 weeks later; Fru-LVD: rats were treated as Group Fru, and low-dose calcitriol (10 ng / 100 g body weight per day) was administered 4 weeks later. Values are means ± SD. * denotes *P* < 0.05 *vs*. pre-fructose period. ^ and # denote *vs*. control rats and fructose-fed rats at the corresponding time point, respectively. N = 6 for each group.

Changes in SBP in the fructose-fed rats with or without calcitriol treatment are shown in Fig. 1. A high-fructose diet caused a significant rise in SBP from the baseline value of 118 ± 4.7 mmHg to 140 ± 5.3 and 148 ± 4.6 mmHg at Day 28 and Day 56, respectively. High- and low-dose calcitriol treatment significantly decreased the SBP in the fructose-fed rats by 14 ± 4 and 9 ± 4 mmHg, respectively, at Day 56.

### High-dose Calcitriol Treatment Mildly Increased Serum Ionized Calcium but Did Not Alter Serum Creatinine in the Fructose-fed Rats

**Fig 2:**
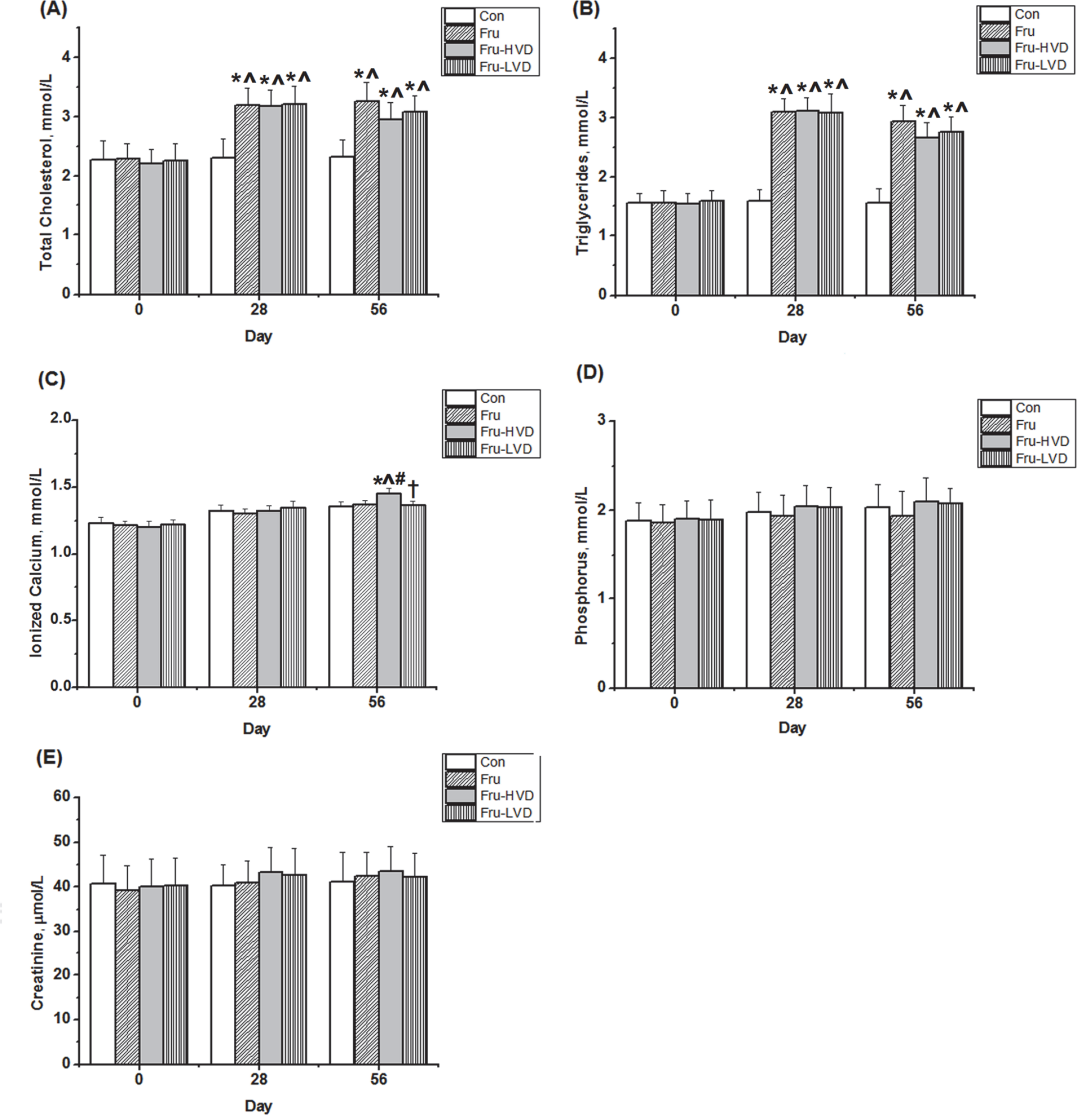
The biochemical effects of calcitriol treatment on the fructose-fed rats: (A) total cholesterol, (B) triglycerides, (C) ionized calcium, (D) phosphorus, and (E) creatinine. Con: control rats with normal chow diet; Fru: rats were fed a high-fructose diet for 8 weeks; Fru-HVD: rats were treated as Group Fru, and high-dose calcitriol (20 ng / 100 g body weight per day) was administered 4 weeks later; Fru-LVD: rats were treated as Group Fru, and low-dose calcitriol (10 ng / 100 g body weight per day) was administered 4 weeks later. Values are means ± SD. * denotes *P* < 0.05 *vs*. pre-fructose period. ^ and # denote *vs*. control rats and fructose-fed rats at the corresponding time point, respectively. † denotes *P* < 0.05 vs. Fru-HVD group at the corresponding time point. N = 6 for each group.

The effects of a high-fructose diet alone and in combination with calcitriol treatment on serum triglycerides, total cholesterol, ionized calcium, phosphorus, and creatinine are summarized in Fig. 2. A high-fructose diet significantly increased serum triglycerides (2.94 ± 0.27 mmol/L) and total cholesterol (3.27 ± 0.31 mmol/L) levels at Day 56 compared with the control rats. High- and low-dose calcitriol treatment did not improve the levels of serum triglycerides or total cholesterol in the fructose-fed rats. In addition, high-dose calcitriol treatment (20 ng/kg per day) significantly increased serum ionized calcium level (1.44 ± 0.05 mmol/L) compared with the other groups. Serum creatinine, representing renal function, did not change among these four groups throughout the course of the study. Additionally, serum 1,25-(OH)_2_D_3_ levels were decreased in fructose-fed rats; further, both physiological dosages of calcitriol treatment ameliorated serum 1,25-(OH)_2_D_3_ levels in fructose-fed rats, as the Table 1.

**Table 1:** Effects of high fructose diet alone and in combination with calcitriol treatment on 1,25-(OH)_2_D_3_ and insulin concentrations.

Group	Day 0				Day 28				Day 56			
	Con	Fru	Fru-HVD	Fru-LVD	Con	Fru	Fru-HVD	Fru-LVD	Con	Fru	Fru-HVD	Fru-LVD
N	6	6	6	6	6	6	6	6	6	6	6	6
1,25-(OH)_2_D_3_, (pmol/L)	490±65	488±56	496±62	502±55	484±50	340±45* ^^^	336±52* ^^^	348±58* ^^^	498±58	328±56* ^^^	492±52^#^	480±60^#^
Insulin, (pmol/L))	156±24	152±27	158±25	155±26	153±22	296±30* ^^^	290±27* ^^^	292±32* ^^^	155±26	309±28* ^^^	220±33* ^^^ ^#^	248±26* ^^^ ^#^

Con: control rats with normal chow diet; Fru: rats were fed a high-fructose diet for 8 weeks; Fru-HVD: rats were treated as Group Fru, and high-dose calcitriol (20 ng / 100 g body weight per day) was administered 4 weeks later; Fru-LVD: rats were treated as Group Fru, and low-dose calcitriol (10 ng / 100 g body weight per day) was administered 4 weeks later. Values are means ± SD.

* denotes *P* < 0.05 *vs*. Day 0

^ and # denote *P* < 0.05 *vs*. control rats and fructose-fed rats at the corresponding time point, respectively.

### Calcitriol Improved Serum Glucose and IGTT in the Fructose-fed Rats

**Fig 3:**
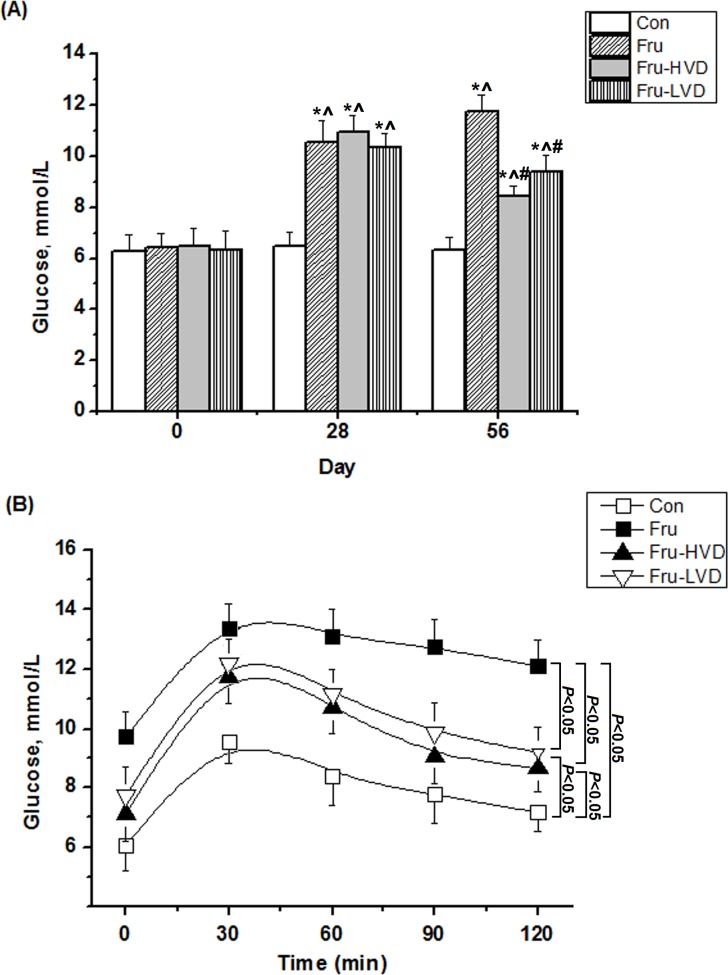
Effects of calcitriol on (A) glucose and (B) blood glucose curves following intra-peritoneal glucose loading in the fructose-fed rats. Con: control rats with normal chow diet; Fru: rats were fed a high-fructose diet for 8 weeks; Fru-HVD: rats were treated as Group Fru, and high-dose calcitriol (20 ng / 100 g body weight per day) was administered 4 weeks later; Fru-LVD: rats were treated as Group Fru, and low-dose calcitriol (10 ng / 100 g body weight per day) was administered 4 weeks later. Values are means ± SD. * denotes *P* < 0.05 *vs*. pre-fructose period. ^ and # denote *vs*. control rats and fructose-fed rats at the corresponding time point, respectively. N = 6 for each group.

The effects of calcitriol on serum glucose and IGTT in the fructose-fed rats are shown in Fig. 3. Serum glucose levels in the fructose-fed rats were significantly higher than those in the control rats at Day 28 and Day 56. However, high- and low-dose calcitriol treatment significantly decreased the serum glucose level in the fructose-fed rats by 3.32 and 2.36 mmol/L, respectively, at Day 56 (Fig. 3A). For serum insulin levels, a high fructose diet caused the rise of serum insulin levels; further, both physiological dosages of calcitriol treatment diminished serum insulin levels in fructose-fed rats (Table 1). Moreover, the response of serum glucose levels to intra-peritoneal glucose loading was significantly greater in the fructose-fed rats (from 9.73 ± 0.85 to 12.10 ± 0.89 mmol/L) than those in the control rats (from 6.05 ± 0.83 to 7.15 ± 0.90 mmol/L), as Fig. 3B. Compared with the fructose-fed rats group, high- and low-dose calcitriol treatments for fructose-fed rats had significantly ameliorated the response of serum glucose levels to intra-peritoneal loading (from 7.09 ± 0.87 to 8.64 ± 0.87 mmol/L, and from 7.72 ± 0.96 to 9.21 ± 0.88 mmol/L, respectively).

### Calcitriol Improved Endothelium-dependent Vascular Relaxation in the Aortas of the Fructose-fed Hypertensive Rats

**Fig 4:**
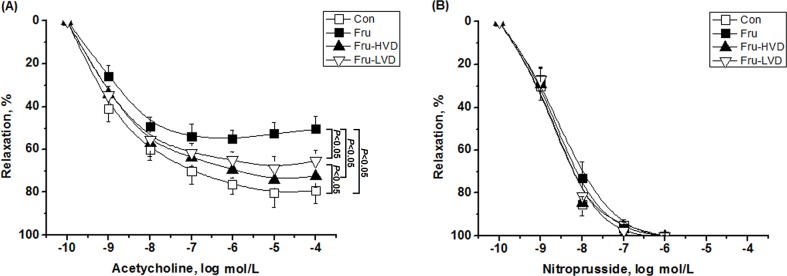
Effects of calcitriol on *in vitro* vascular relaxation of the fructose-fed rats. (A) Endothelium-dependent vascular relaxation in response to acetylcholine and (B) endothelium-independent vascular relaxation in response to sodium nitroprusside in thoracic aortic segments. Con: control rats with normal chow diet; Fru: rats were fed a high-fructose diet for 8 weeks; Fru-HVD: rats were treated as Group Fru, and high-dose calcitriol (20 ng / 100 g body weight per day) was administered 4 weeks later; Fru-LVD: rats were treated as Group Fru, and low-dose calcitriol (10 ng / 100 g body weight per day) was administered 4 weeks later. Vessels were studied as ring segments in organ chambers, and relaxations in response to acetylcholine and sodium nitroprusside were measured. Values are means ± SD. N = 6 for each group.

The effects of calcitriol on relaxing responses to acetylcholine and sodium nitroprusside in the fructose-fed rats with or without calcitriol treatment are shown in Fig. 4. The peak aortic relaxation induced by acetylcholine in the fructose-fed rats was significantly reduced compared with the control rats. The peak aortic relaxation in response to acetylcholine in the fructose-fed rats with high- or low-dose calcitriol treatment was significantly larger than in the fructose-fed rats without calcitriol treatment (74.46 ± 4.08 and 68.76 ± 5.28% versus 52.49 ± 5.25%, respectively; *P* < 0.05). High-dose calcitriol treatment in the fructose-fed rats restored acetylcholine-dependent aortic relaxation to that of the control rats, however low-dose calcitriol treatment did not.

### Calcitriol Reduced the Weight of Visceral Fat Pads and Visceral Adipose Sizes in the Fructose-fed Rats

**Fig 5:**
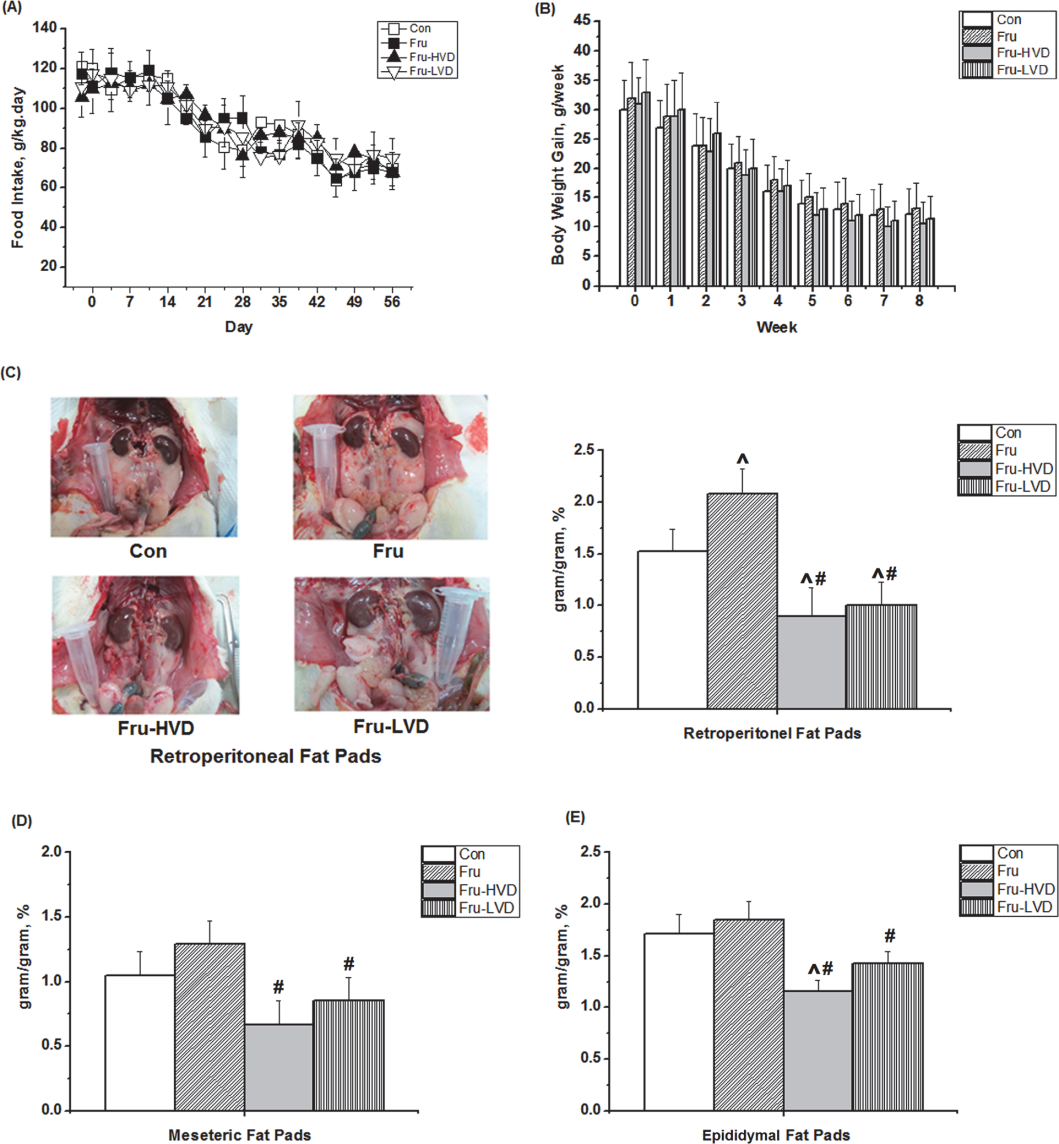
Effects of calcitriol on (A) daily food intake, (B) body weight, (C) ratio of retroperitoneal fat pad weight over body weight (gram/gram, %), (D) ratio of mesenteric fat pad weight over body weight (gram/gram, %), and (E) ratio of epididymal fat pad weight over body weight (gram/gram, %) in the fructose-fed rats. Con: control rats with normal chow diet; Fru: rats were fed a high-fructose diet for 8 weeks; Fru-HVD: rats were treated as Group Fru, and high-dose calcitriol (20 ng / 100 g body weight per day) was administered 4 weeks later; Fru-LVD: rats were treated as Group Fru, and low-dose calcitriol (10 ng / 100 g body weight per day) was administered 4 weeks later. Values are mean ± SD. N = 6 for each group. ^ and # denote vs. control rats and fructose-fed rats at the corresponding time point, respectively. N = 6 for each group.

**Fig 6:**
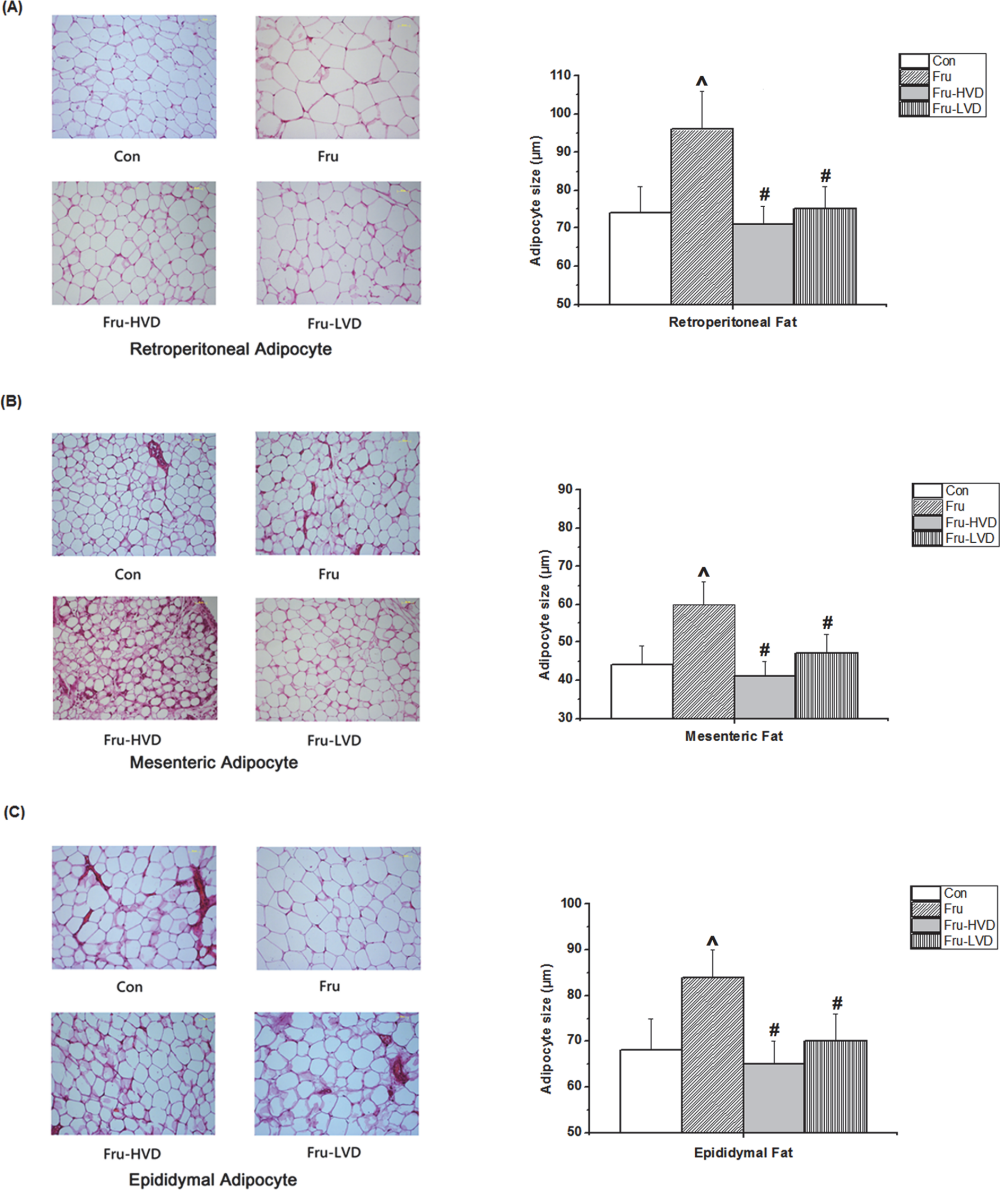
Effects of calcitriol on the sizes of adipocytes from (A) retroperitoneal fat, (B) mesenteric fat, and (C) epididymal fat in the fructose-fed rats. Con: control rats with normal chow diet; Fru: rats were fed a high-fructose diet for 8 weeks; Fru-HVD: rats were treated as Group Fru, and high-dose calcitriol (20 ng / 100 g body weight per day) was administered 4 weeks later; Fru-LVD: rats were treated as Group Fru, and low-dose calcitriol (10 ng / 100 g body weight per day) was administered 4 weeks later. Original magnification × 40. Values are means ± SD. N = 6 for each group. ^ and # denote *vs*. control rats and fructose-fed rats at the corresponding time point, respectively. N = 6 for each group.

The effects of calcitriol on daily food intake, body weight, and ratio of visceral fat pads weight over whole body weight (gram/gram, %) in the fructose-fed rats are presented in Fig. 5. Daily food intake and body weight did not differ among the four groups at the corresponding time points throughout the entire study period. Compared with the control rats, the ratio of the retroperitoneal fat pads weight over whole body weight (gram/gram, %) was higher in the fructose-fed rats (2.08 ± 0.24% vs. 1.53 ± 0.21%; *P* < 0.05). Further, the ratio of the retroperitoneal, mesenteric, and epididymal fat pads weight over whole body weight (gram/gram, %) were reduced by either high- or low-dose calcitriol treatment compared to the fructose-fed rats without calcitriol treatment. Besides, the ratio of the other visceral organs weight over whole body weight (gram/gram, %) was not significantly different among these group, as S1 Table. The mean sizes of adipocytes derived from the fat pads in the fructose-fed rats were noticeably larger than those in the control rats (Fig. 6). Both low- and high-dose calcitriol treatment significantly reduced the adipocyte sizes of the retroperitoneal, mesenteric, and epididymal fat pads in the fructose-fed rats.

### Calcitriol Reduced Adipose Ang II Levels from Visceral Adipose Tissues in the Fructose-fed Rats

**Fig 7:**
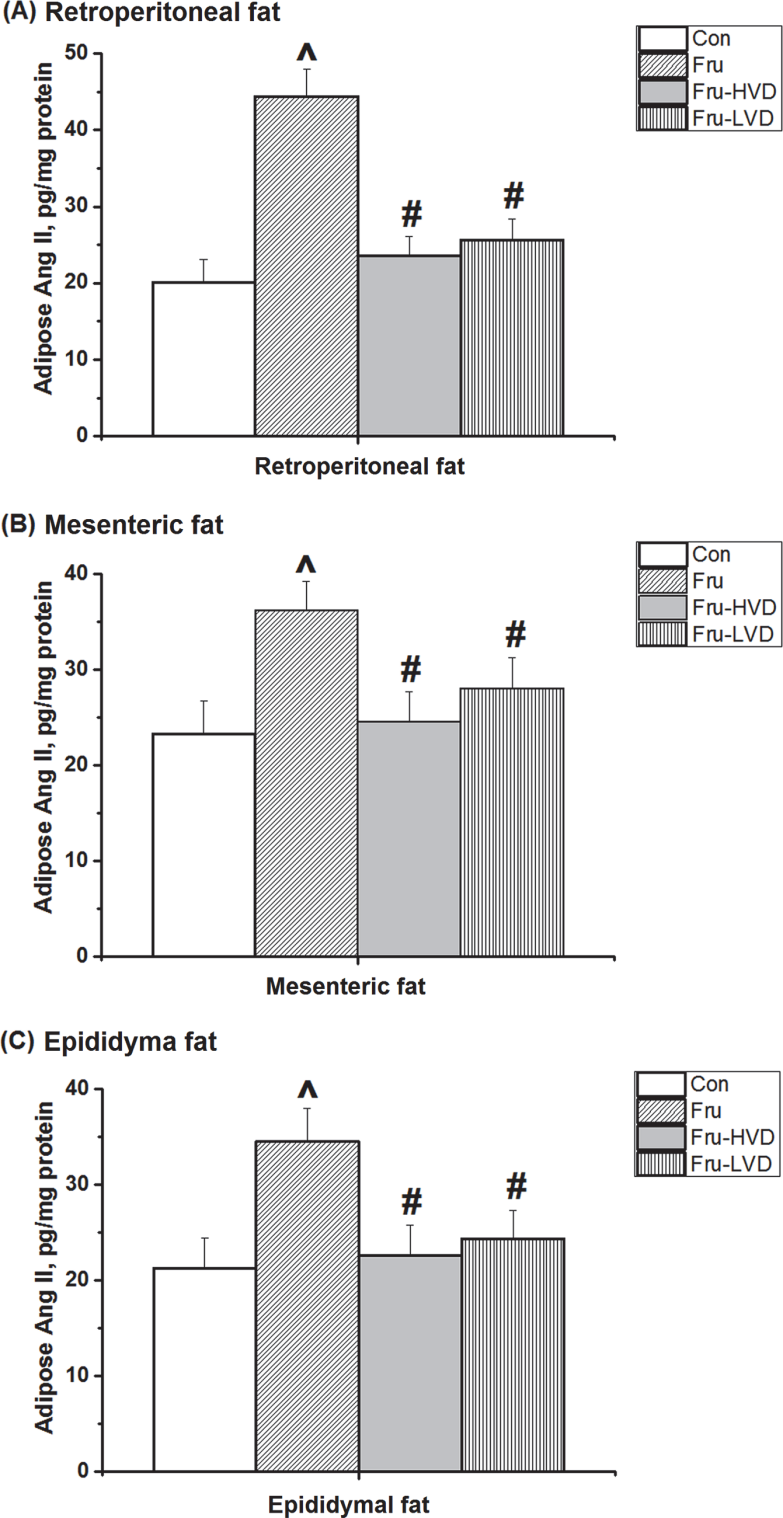
Effects of calcitriol on adipose angiotensin II (Ang II) concentration in (A) retroperitoneal fat, (B) mesenteric fat, and (C) epididymal fat in the fructose-fed rats. Con: control rats with normal chow diet; Fru: rats were fed a high-fructose diet for 8 weeks; Fru-HVD: rats were treated as Group Fru, and high-dose calcitriol (20 ng / 100 g body weight per day) was administered 4 weeks later; Fru-LVD: rats were treated as Group Fru, and low-dose calcitriol (10 ng / 100 g body weight per day) was administered 4 weeks later. Values are mean ± SD. N = 6 for each group. ^ and # denote vs. control rats and fructose-fed rats at the corresponding time point, respectively. N = 6 for each group.

Fig. 7 shows the effect of calcitriol on adipose Ang II levels derived from retroperitoneal, mesenteric, and epididymal fat pads. A high-fructose diet caused an increase in Ang II expression from the visceral adipose tissues of the rats. High- or low-dose calcitriol treatment in the fructose-fed rats reduced the raised Ang II expressions from the visceral adipose tissues including retroperitoneal, mesenteric, and epididymal fat pads.

## Discussion

In this model of fructose-fed hypertensive rats, we examined the effect of calcitriol, an active form of vitamin D, on SBP, biochemistry, endothelium-dependent vascular relaxation, and fat tissue in fructose-fed rats. We showed that a high-fructose diet in the rats caused systolic hypertension, increased serum levels of glucose, triglycerides, and total cholesterol, and increased the weight of visceral fat pads, which is consistent with previous studies [37–41]. Furthermore, the major findings of this study are that: (1) high- or low-dose calcitriol treatment reduced SBP and partially improved the impairment of endothelium-dependent aortic relaxation in the fructose-fed rats; (2) high- or low-dose calcitriol treatment reduced serum glucose levels and improved glucose intolerance in the fructose-fed rats, however high-dose calcitriol led to a modest but significant hypercalcemia compared with the other 3 groups; (3) a high-fructose diet increased the weight of retroperitoneal, mesenteric, and epididymis fat pads and adipose sizes, and high- or low-dose calcitriol treatment significantly decreased these changes in the fat tissues; and (4) a high-fructose diet increased adipose Ang II expressions in the visceral adipose tissues, and high- or low-dose calcitriol treatment reduced the increase in Ang II expressions in retroperitoneal, mesenteric, and epididymal fat. Our results suggest that calcitriol plays a key role in the regulation of blood pressure, vascular relaxation, glucose tolerance, and the weight of visceral fat pads and adipose sizes through down-regulation of RAS in fructose-fed rats.

Whether calcitriol reduced the SBP in the fructose hypertensive rats is unknown, although previous studies have shown that calcitriol is an effective renin inhibitor [16, 17, 42], and other studies have shown its antihypertensive efficacy in SHR and renovascular hypertensive rats through inhibiting RAS activation [18, 21]. For example, the SBP in SHR was significantly reduced by 25 mmHg after calcitriol treatment with the dosage of 15 ng / 100 g per day [21]. In addition, the other study reported that the mean arterial blood pressure in SHR treated with calcitriol treatment with 10 ng / 100 g per day was significantly lowered from 198.3 ± 5.4 mmHg to 165.6 ± 12.8 mmHg [18]. Similarly, our results showed that high-dose (20 ng / 100g per day) or low-dose (10 ng / 100 g per day) calcitriol treatment decreased the SBP of fructose-fed rats by 14 ± 4 and 9 ± 4 mmHg, respectively. As previously reported, the blood pressure lowering properties of calcitriol may be mediated through inhibiting renin activation and subsequently down-regulating Ang II type 1 receptors and reducing oxidative stress [21]. Furthermore, Freundlich *et al*. demonstrated that beneficial effects of vitamin D treatment are related to suppression of renin-angiotensin gene expression with decreasing mRNA levels of angiotensinogen, renin, and renin receptor [42]. Similarly, current clinical observational studies strongly support an inverse association between the level of vitamin D and blood pressure [5]. This inverse relationship has also been observed in Vitamin D receptor (VDR) knockout mice [16] and in 1-alphahydroxylase knockout mice [43]. Additionally, some randomized, controlled trials have also reported a beneficial effect of vitamin D on lowering diastolic blood pressure in studies of hypertensive patients [44]. Taken together, these results indicate a possible role of vitamin D in the regulation of blood pressure.

Numerous studies have reported that raised blood pressure is characterized by endothelial dysfunction mediated by impaired NO availability [45, 46]. However, it remains unclear whether calcitriol supplementation can improve endothelial function in hypertensive animals. In this study, we administered calcitriol (10 or 20 ng / 100 g per day) to fructose-fed rats, and the results showed that calcitriol improved the impairment of endothelium-dependent aortic relaxation in the fructose-fed rats, possibly by stimulating NO release. This result is similar to studies on the effects of calcitriol on endothelium-dependent vascular relaxation of SHR experiments, which showed that calcitriol treatment (10 ng/100 g per day) could increase endothelium-dependent relaxation in the aorta [18] or renal artery [21]. Interestingly, Dong et al. also showed that in normal rats with standard chow diets, calcitriol treatment at the physiologic dosage of 10 ng / 100 g body weight per day has not affected blood pressure and vascular relexation [18]. Further, *in vitro*, the impaired endothelium-dependent vascular relaxation in SHR has been shown to be partially rescued by 12-hour incubation with calcitriol, suggesting that calcitriol has a direct effect on improving vascular tone [19, 21].

Glucose metabolism is a concern as an extra-skeletal function of vitamin D. Some clinically studies have shown an association between vitamin D deficiency and impaired glucose tolerance [47, 48]. Similarly, a previous animal study showed that vitamin D deficiency or VDR knockout impaired the ability of insulin secretion and induced hyperglycemia [49]. Further, with regards to whether vitamin D supplementation can improve glucose intolerance, our results showed that high- or low-dose calcitriol treatment improved hyperglycemia and glucose intolerance in the fructose-fed rats. Intra-peritoneal glucose test is a reliable test and has been reported in some studies [34, 50, 51]. Besides, some studies also reported that vitamin D treatments could reduce insulin levels and ameliorate insulin resistance in experimental diabetes models [52, 53]. Emerging evidences suggest that vitamin D supplementation may have beneficial effects on improving glucose intolerance [9]. Previous studies have reported that the mechanisms by which vitamin D treatment improves glucose intolerance include: (1) the stimulation of insulin secretion through lessening pancreatic RAS activation [54]; and (2) increasing insulin sensitivity via raising insulin receptor transcription [55]. Thus, further studies are required to confirm the effect of vitamin D on glucose metabolism.

Regarding the effect of high fructose diet on serum vitamin D levels, the results of serum 1,25-(OH)_2_ D_3_ levels in our study resembled Douard *et al*. report [35], showing fructose-fed diet caused a 30–40% decrease in serum 1,25-(OH)_2_ D_3_ levels in rats. The study from Douard et al. [35] presented that a 63% fructose diet in rats decreased circulating 1,25-(OH)_2_ D_3_ levels because of lessened 1α-hydroxylase expression and elevated fibroblast growth factor 23, and further diminished intestinal and renal Ca^2+^ transporter expression, but did not affect circulating levels of calcium, phosphorus, and parathyroid hormone. Additionally, Douard’s study further observed that decreasing serum 1,25-(OH)_2_D_3_ levels following a very high dosage (100 ng/ 100 g body weight per day) of calcitriol treatment probably was because using this very high dosage inhibited its own synthesis of endogenous 1,25-(OH)_2_D_3_ by reducing CYP27B1 expression and ensuring the rapid clearance of serum 1,25-(OH)_2_D_3_ by increasing CYP24A1 expression [35]. However, Saito *et al*. demonstrated that the physiological dosage of calcitriol treatments in rats (less than 25 ng/ 100 g body weight per day) did not influence CYP27B1 and CYP24A1 expression [56]. Therefore, both physiological dosages of calcitriol treatments in our study could ameliorate serum 1,25-(OH)2D3 levels in fructose-fed rats, and this result might be due to physiological dosages of calcitriol treatments having no inhibition on the synthesis of endogenous 1,25-(OH)2D3. Therefore, this fructose-induced hypertensive models could be regarded as a vitamin D deficient-induced hypertensive model. Also, our study presented that calcitriol treatments at a physiologic dosage of either 10 or 20 ng / 100 g body weight per day had beneficial effects on systolic blood pressure, glucose intolerance, and endothelium-dependent aortic relaxation.

Concerning the concept of safety for daily vitamin D supplement, a physiologic dosage of daily vitamin D intake poses no risks of adverse health effects to almost all individuals in the general population [57]. The reason is that the serum 25(OH) Vitamin D concentration is maintained within an optimal and harmless range during administering a daily physiologic dosage of vitamin D [58]. For a physiologic dosage of vitamin D in the rats, calcitriol treatments at less than 25 ng / 100 g body weight per day does not cause any adverse effects, such as hypercalcemic hypercalciuria [22], renal dysfunction [23], and vascular calcifications [59]. For example, in normal rats with standard chow diets, caclitriol treatment at 25 ng/kg body weight per day for 14 days did not induce hyperphosphtemic hyperphosphturia and hypercalcemic hypercaluria with an optimal vitamin D range [22]. Recently, calcitriol treatments through down-regulation of RAS have been reported to improve aortic vascular tone and cardiac hypertrophy in SHR [18–20] as well as ameliorate pancreatic islets dysfunction under high-glucose stress in isolated islets [54]. In our study, fructose-fed rats were respectively treated by calcitriol at 10 or 20 ng/ 100 g body weight per day, which were below an upper limitation of physiologic dosage (less than 25 ng/ 100 g body weight per day) and harmless for rats. Moreover, compared with calcitriol group at 10 ng / 100 g body weight per day, we observed a modest but significant elevation of serum ionized calcium (1.44 ± 0.05 mmol/L) in calcitriol group at 20 ng / 100 g body weight per day, which is consistent with a previous study that showed calcitriol treatment at a dose of 20 ng / 100 g per day for 28 days in healthy rats with a standard diet led to a mild elevation of serum calcium levels without any adverse effects [60]. However, it has been reported that rats receiving intra-peritoneal injections of very high calcitriol (100 ng / 100 g body weight per day) had medial artery calcification of the aorta without affecting renal function at day 8, and reversibility of the artery calcification after withdrawal of calcitriol treatment [61]. Besides, Atchison et al. also showed that a high dosage of calcitriol treatment (100 ng per day) induced hypercalcemic hypercalciuria with volume depletion to trigger RAS activity rather than inhibit RAS activity in normal rats [62].

To the best of our knowledge, this is the first study on the effect of calcitriol on the fat tissues of fructose-fed rats. Our results showed that a high-fructose diet increased the weight of retroperitoneal, mesenteric, and epididymal fat pads and adipose sizes in the rats, and that calcitriol treatment decreased these changes. In addition, a high-fructose diet increased Ang II levels in the retroperitoneal, mesenteric, and epididymal fat pads of the rats, and subsequent calcitriol treatment then lowered these Ang II levels. Further, calcitriol treatment reduced the weights of the visceral fat pads and adipose sizes through down-regulation of adipose RAS. Several previous studies have demonstrated that a fructose diet in rats could increase the weight of visceral fat pads, and subsequent inhibition of the RAS activity could reduce the weight of visceral fat pads and adipose sizes [13, 37, 63]. For example, rats fed a fructose diet were shown to have increased adipose expressions of type 1 Ang II receptors via the up-regulation of RAS [13] accompanied by heavier weights of epididymal fat pads and adipocyte sizes [37]. Subsequently, blockade of RAS lessened the enlarged adipocyte sizes by both temocapril, an angiotensin-converting enzyme inhibitor, and olmesartan treatment, an Ang II type 1 receptor blocker [37]. Moreover, aliskiren treatment, a renin inhibitor, has been shown to reduce whole body weight and the weight of visceral fat pads in fat-fed rats [63]. These findings suggest that RAS may play a role in regulating visceral adiposity in fructose-fed rats. In addition, epidemiological studies have reported an inverse relationship between vitamin D status and obesity in healthy adults [64, 65]. Thus, the regulation of vitamin D in adiposity through RAS merits further investigations.

Concerning adipose RAS effects, the up-regulation of adipose RAS by fructose-feeding promotes inflammation and reactive oxygen species generation. Then, the adipose RAS activity modulates systemic RAS activity, and subsequently develops metabolic syndrome [66]. Therefore, the detection of adipose RAS, not circulating RAS, has been suggested as an appropriate way to evaluate RAS activity [13, 21, 42, 54]. For example, Hwang et al. showed that a high fructose diet caused hypertension, glucose intolerance, hyperlipidemia, but there were no significant changes in PRA throughout the experiment [67]. Afterwards, Giacchetti et al. suggested that raised blood pressure levels in high fructose diets could be related to up-regulation of adipose RAS [13].

In conclusion, our data suggest that fructose-fed rats exhibit significantly increased hypertension and other metabolic manifestations, such as increased serum glucose, triglyceride, and total cholesterol levels. Both low- and high-dose calcitriol treatment could improve glucose intolerance, reduce hypertension, improve the impairments of endothelium-dependent vascular relaxation, decrease the enlargement of visceral fat pads and adipose sizes of visceral fat pads, and reduce the elevation of adipose Ang II expression in fructose-fed rats. However, high-dose calcitriol treatment a modest but significantly increased serum ionized calcium levels compared with low-dose calcitriol treatment. Taken together, these findings suggest a protective role of calcitriol in endothelial function, glucose tolerance, and visceral adiposity in fructose-fed rats. Calcitriol-related benefits merit further investigations due to the worldwide increase in intake of high-fructose corn syrup.

## Supporting Information

S1 Table Effects of a high-fructose diet, alone and in combination with calcitriol treatment, on the ratio of organs weight over whole body weight (gram/gram, %). (DOC)
