# Supplementary material for: Beneficial Effects of Calcitriol on Hypertension, Glucose Intolerance, Impairment of Endothelium-Dependent Vascular Relaxation, and Visceral Adiposity in Fructose-Fed Hypertensive Rats
Source: PLoS One. 2015 Mar 16;10(3):e0119843. doi: 10.1371/journal.pone.0119843 (PMC4361671; doi:10.1371/journal.pone.0119843)
Supplement: S1 Table — (DOC) [file pone.0119843.s001.doc]

**Supplement Table.** Effects of a high-fructose diet, alone and in combination with calcitriol treatment, on the ratio of organs weight over whole body weight (gram/gram, %)

|  | N | BW  (g) | Heart  (g/g BW, %) | Liver  (g/g BW, %) | Right kidney  (g/g BW, %) | Left kidney  (g/g BW, %) | Pancreas  (g/g BW, %) |
| --- | --- | --- | --- | --- | --- | --- | --- |
| Con | 6 | 309.34±15.12 | 0.37±0.24 | 2.89±0.41 | 0.46±0.05 | 0.46±0.08 | 0.56±0.05 |
| Fru | 6 | 320.06±18.56 | 0.41±0.04 | 3.35±0.33 | 0.49±0.06 | 0.49±0.04 | 0.48±0.05 |
| Fru-HVD | 6 | 287.17±19.92 | 0.38±0.12 | 2.98±0.43 | 0.48±0.04 | 0.47±0.06 | 0.53±0.04 |
| Fru-LVD | 6 | 295.72±16.28 | 0.39±0.11 | 3.21±0.34 | 0.48±0.09 | 0.49±0.08 | 0.52±0.04 |

Con: control rats with normal chow diet; Fru: rats were fed a high-fructose diet for 8 weeks; Fru-HVD: rats were treated as Group Fru, and high-dose calcitriol (20 ng / 100 g body weight per day) was administered 4 weeks later; Fru-LVD: rats were treated as Group Fru, and low-dose calcitriol (10 ng / 100 g body weight per day) was administered 4 weeks later ; BW, body weight; g/g, gram/gram. Values are means ± SD.
